# Spike reliability is cell type specific and shapes excitation and inhibition in the cortex

**DOI:** 10.1038/s41598-024-82536-y

**Published:** 2025-01-02

**Authors:** Simone Russo, Garrett B. Stanley, Farzaneh Najafi

**Affiliations:** 1 Wallace H Coulter Department of Biomedical Engineering, Georgia Institute of Technology and Emory University, 313 Ferst Dr NW, GA 30332-0535 Atlanta, USA; 2 Allen Institute, Brain and Consciousness Program, Seattle, WA USA; 3 School of Biological Sciences, Georgia Institute of Technology, 315 Ferst Dr NW, Atlanta, 30332-0535 GA USA

**Keywords:** Reliability, Variability, Parvalbumin neurons, Excitatory neurons, Spike timing precision, Cellular neuroscience, Neural circuits, Neuronal physiology

## Abstract

Neurons encode information in the highly variable spiking activity of neuronal populations, so that different repetitions of the same stimulus can generate action potentials that vary significantly in terms of the count and timing. How does spiking variability originate, and does it have a functional purpose? Leveraging large-scale intracellular electrophysiological data, we relate the spiking reliability of cortical neurons in-vitro during the intracellular injection of current resembling synaptic inputs to their morphologic, electrophysiologic, and transcriptomic classes. Our findings demonstrate that parvalbumin+ (PV) interneurons, a subclass of inhibitory neurons, show high reliability compared to other neuronal subclasses, particularly excitatory neurons. Through computational modeling, we predict that the high reliability of PV interneurons allows for strong and precise inhibition in downstream neurons, while the lower reliability of excitatory neurons allows for integrating multiple synaptic inputs leading to a spiking rate code. These findings illuminate how spiking variability in different neuronal classes affect information propagation in the brain, leading to precise inhibition and spiking rate codes.

**Supplementary Information:**

The online version contains supplementary material available at 10.1038/s41598-024-82536-y.

## Introduction

Neurons encode continuously varying information through discrete action potentials. Action potentials have low reliability, so that neurons respond to multiple repetitions of the same stimulus with a different number of action potentials and at different timings^1–7^. This leads to a large variability across repetitions, that is usually discarded through averaging while preserving reliable neural responses, invariant across repetitions.

Over the last few decades, variability in neural activity has been progressively re-evaluated; from first being a nuisance, to later becoming a valuable resource. Recent studies postulated that variability in neuronal activity is essential for behaviors such as discrimination^8,9^, encodes important sensory and behavioral features^10–16^, and promotes efficient information coding^17,18^. The new understanding of the importance of neuronal reliability (and its opposite, variability) prompted further studies to identify its origins. These studies found that cortical responses to sensory stimuli are unreliable^19^ due to independent inputs, both from the environment or from the network^1,20–22^. One intriguing hypothesis is that distinct subtypes of cortical neurons may have distinct reliability properties, and their coordinated activity defines the overall reliability of the cortex in response to external stimuli^23^. Indeed, previous studies found that inhibitory neurons may respond more reliably to external stimuli compared to excitatory neurons^24,25^, thus suggesting that different cell types may exhibit different levels of reliability due to their membrane properties^26^, but this has not been explicitly tested.

Unraveling reliability in distinct neuronal subtypes is crucial to understanding how they interact to process information in neural circuits. We address this question by investigating spiking reliability across stimuli through measures that incorporate both spike occurrence and temporal precision^27^. Specifically, we studied how reliability across spikes induced by direct neuronal stimulation relates to neuronal cell types, and how it impacts the effect of spikes on downstream neurons.

## Results

We leveraged the Allen Cell Types dataset^28,29^, which characterizes the morphologic, electrophysiologic, and transcriptomic features of mice and human cortical neurons in-vitro. Based on these features, we classified neurons in morphologic (aspiny, sparsely spiny, and spiny dendrites), electrophysiologic (fast and regular spiking), and transcriptomic cell-types (transgenic mouse lines).

**Fig. 1:**
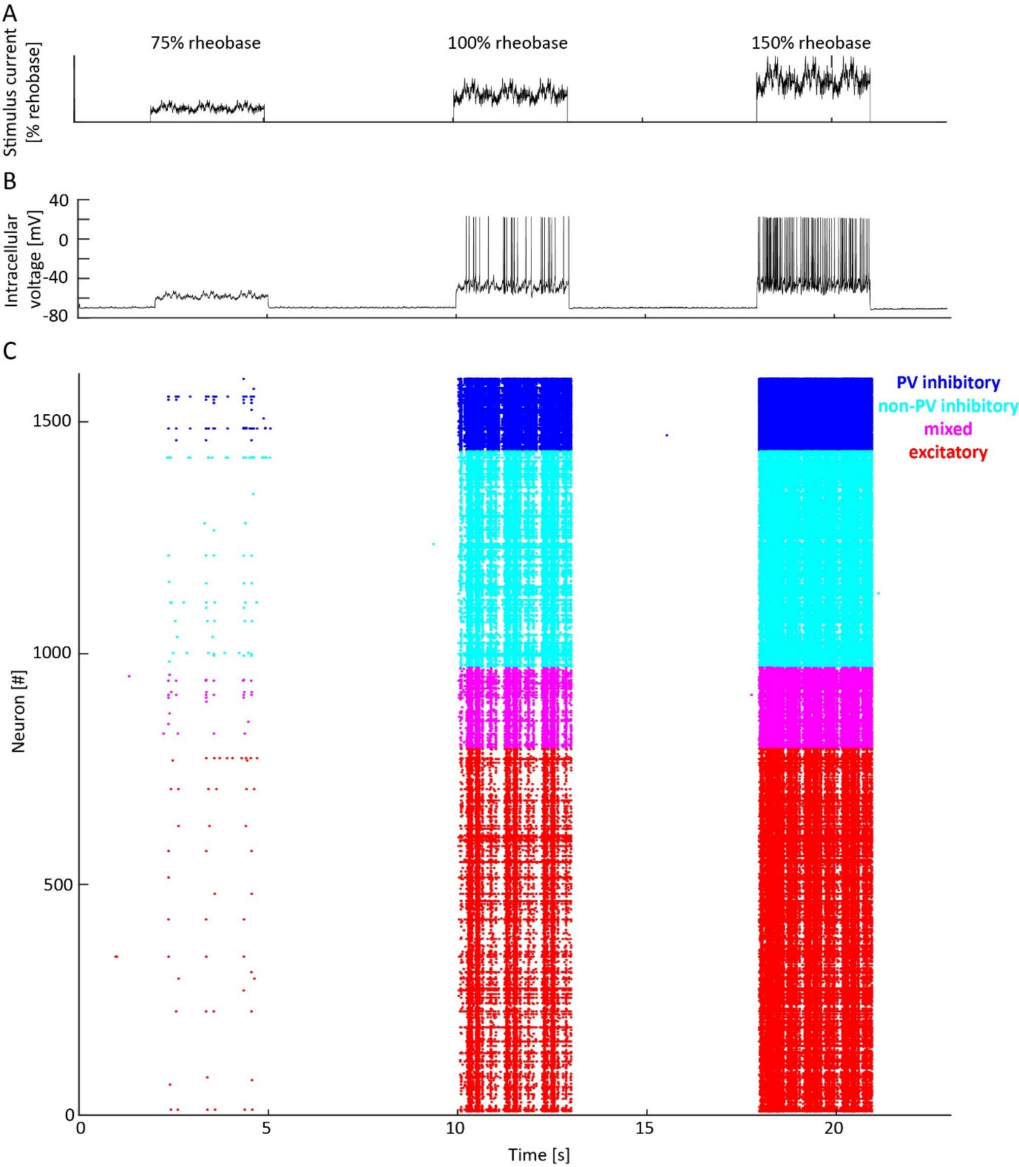
Pink noise current stimulation induces spiking activity in neocortical neurons in the Allen Cell Types dataset^28,29^. (**A**) Time course of Noise 2 current (intensity: 75%, 100%, and 150% rheobase) administered in the stimulation protocol. (**B**) Intracellular voltage of one recording from one representative neuron stimulated with Noise 2. (**C**) Spike timing elicited by Noise 2 in each stimulated mouse neuron. Neurons are color-coded according to the four groups of transcriptomic cell types: PV (parvalbumin) inhibitory, non-PV inhibitory, mixed, and excitatory cell-types.

**Fig. 2:**
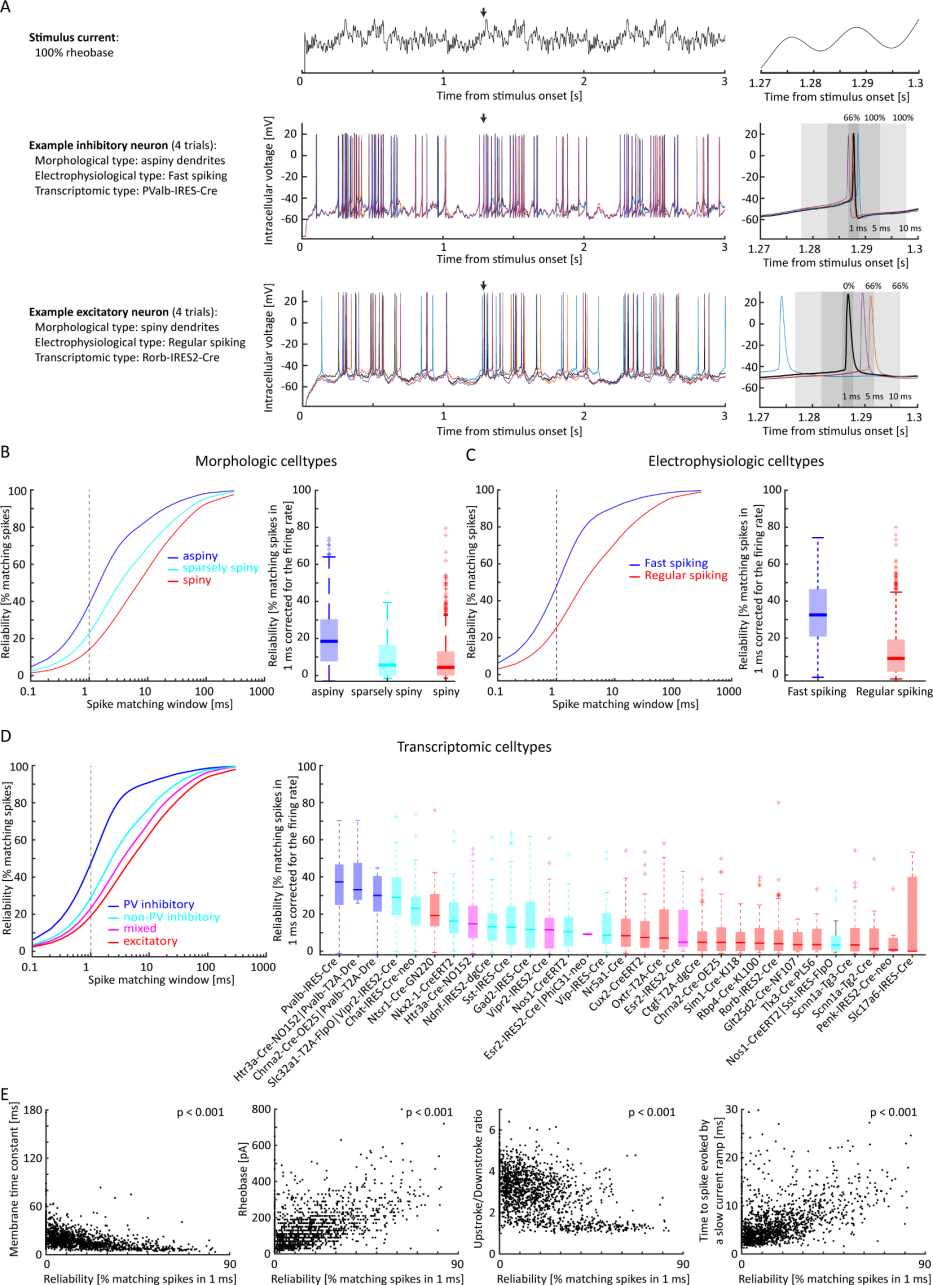
Noise stimulation induces more reliable response in morphologic, electrophysiologic, and transcriptomic cell-types associated with specific inhibitory neuronal populations in mice. (**A**) Trace of the Noise 2 current at 100% rheobase (top). For one representative inhibitory and excitatory neuron (middle and bottom), we (left) summarize their morphologic, electrophysiologic, and transcriptomic cell-type, (center) show the intracellular voltage activity evoked by Noise 2 stimulation at 100% rheobase across 4 trials, and (right) show their reliability in the same time window (indicated by the black arrow in the central panel), as measured by the percentage of spikes falling within windows of increasing size from the selected spike. (**B**) For morphologic cell types, we illustrate the cumulative probability density function of finding matching spikes across repetitions for increasing time windows (left; Noise 1 and 2 stimuli combined; 706 aspiny, 86 sparsely spiny, and 740 spiny neurons), together with the comparison of the reliability distribution across cell-types (right; window = 1 ms, corrected for firing rate; boxplot reports median [thick line], 25° and 75° percentile [box], maximum and minimum within 1.5 interquartile range [whiskers], and outliers [+]). Color code indicates morphologic cell-types associated with excitatory (red) or inhibitory neurons (blue). (**C**) Same representation as B for electrophysiologic cell-types (159 fast spiking and 1358 regular spiking neurons). (**D**) Same representation as B and C for transcriptomic cell-types. The cumulative probability density functions were grouped in PV (parvalbumin) inhibitory, non-PV inhibitory, mixed, and excitatory cell-types for visualization purposes (145 PV, 452 non-PV inhibitory, 172 mixed, and 763 excitatory neurons). (**E**) We illustrate the correlation between spike reliability and the highly correlated neuronal features (left to right: membrane time constant, rheobase, upstroke/downstroke ratio, and time to spike evoked by a slow current ramp).

To evaluate single-neuron spiking reliability, we analyzed patch-clamp recordings of neurons which had their synaptic inputs blocked (bathed with 1 mM kynurenic acid and 0.1 mM picrotoxin) and were directly stimulated with time-varying pink noise current, resembling physiological synaptic inputs usually simulated with white noise^30,31^. To assess spiking reliability in response to the same stimulus across repetitions, frozen noises (Noise 1 and 2) were administered in current-clamp mode scaled at 75%, 100%, and 150% (Fig. 1A) of the rheobase (minimal electric current necessary to elicit a spike in each neuron). We analyzed 1851 neurons in which noise current stimulation was repeated 2–8 times per noise type (median = 3; Fig. 1B: example mouse neuron; Fig. 1C: all mouse neurons) using specific measures invariant to the number of trials (Fig. 2). To account for differences across species, mouse and human neurons were analyzed separately (Fig. 2 and Figure S1).

Figure 2A shows the activity evoked by noise stimulation in representative inhibitory and excitatory mouse neurons. To assess the spiking reliability of each neuron, we focused on the intracellular voltage induced by 100% rheobase noise stimulation and confirmed the results in the 150% rheobase noise stimulation. We measured the percentage of matching spikes as the percentage of spikes in each repetition that occurred within a window of a given size with respect to the spikes in all other repetitions (Fig. 2A, right). To quantify reliability, we assessed the percentage of matching spikes in a window of 1 ms corrected for firing rate (100 permutations).

For morphologic cell-types, we found that aspiny neurons exhibit more reliable spiking activity compared to spiny and sparsely spiny neurons (Fig. 2B, left). Indeed, aspiny neurons showed significantly higher reliability than spiny and sparsely spiny neurons (Fig. 2B, right; Kruskal-Wallis test; *p* < 0.001; Table S1; Kolmogorov-Smirnov test, *p* < 0.001).

Similarly, for electrophysiological cell-types, fast spiking neurons showed significantly higher reliability than regular spiking neurons (Fig. 2C; Kruskal-Wallis test, *p* < 0.001; Table S1; Kolmogorov-Smirnov test, *p* < 0.001).

Importantly, aspiny dendrites and fast spiking activity are typical of PV inhibitory neurons, while spiny dendrites and regular spiking activity are typical of excitatory neurons and non-PV inhibitory neurons^28^.

Therefore, we analyzed neurons categorized according to their transcriptomic cell-types, and grouped them for visualization purposes in PV inhibitory, non-PV inhibitory, mixed, and excitatory neurons^32^. We found that PV neurons show higher reliability than other neurons (Fig. 2D; Kruskal-Wallis test, *p* < 0.001; Table S1; p-values for Kolmogorov-Smirnov test illustrated in Table S2). For all categorizations, we verified that the stimulation at 150% rheobase intensity led to similar results.

To investigate the origins of spiking reliability, we measured its correlation with other neuronal properties. First, we quantified subthreshold reliability as the Spearman’s correlation of voltages across stimulus repetitions at 75% rheobase. To this aim, we selected the voltage in the time during which the neuron was stimulated with 75% rheobase intensity in each repetition, computed the Spearman’s correlation across each pair of repetitions, and averaged the Spearman’s Rho across all pairs of repetitions from the same neuron. We found overall high reliability in subthreshold fluctuations^33^, but weak correlation between spiking and subthreshold reliability (Spearman’s correlation; Figure S2, *r* = 0.11; *p* < 0.001), suggesting that subthreshold reliability does not drive spiking reliability. Spiking reliability was highly correlated with other intrinsic neuronal features (Fig. 2E) such as membrane time constant (Spearman’s correlation; *r*=-0.61; *p* < 0.001), rheobase (Spearman’s correlation; *r* = 0.55; *p* < 0.001), time to first spike evoked by a slow current ramp (Spearman’s correlation; *r* = 0.49; *p* < 0.001), and ratio between action potential peak upstroke and downstroke (Spearman’s correlation; *r*=-0.40; *p* < 0.001). Interestingly, the correlation between spiking reliability and the membrane time constant was contributed by significant correlations with both membrane capacitance (Spearman’s correlation: *r*=-0.39; *p* < 0.001) and membrane conductance (Spearman’s correlation: *r* = 0.27; *p* < 0.001). Altogether, these findings suggest that spiking reliability may be shaped by the same properties that shape action potentials, such as voltage-dependent sodium and potassium channels, and cell surface area^26,34,35^.

Next, we studied spiking reliability in neurons derived from human samples. Similar to mouse neurons, aspiny and fast spiking neurons (typically associated with PV inhibitory neurons) showed higher spiking reliability; however, human neurons showed overall lower spiking reliability than mouse neurons (Figure S1, 2-way ANOVA *p* < 0.001).

**Fig. 3:**
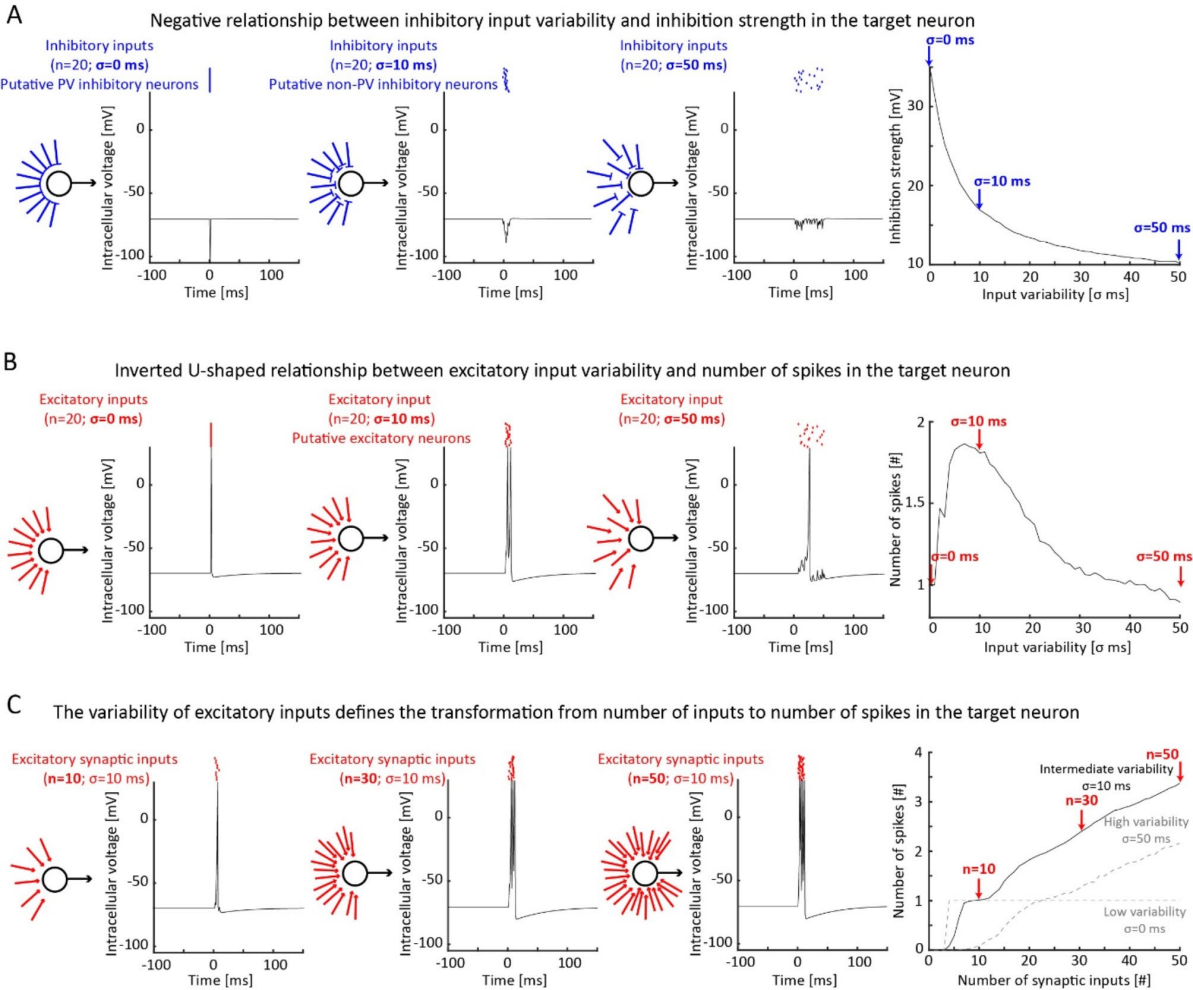
Computational modeling shows that the high reliability of inhibitory inputs increases the strength of the inhibition in downstream neurons while intermediate variability of excitatory inputs can increase the number of spikes generated in downstream neurons. (**A**) From left to right: Schematic of the model showing the administration of inhibitory inputs to the downstream neuron and intracellular voltage evoked in the downstream neuron by 20 inhibitory inputs with low (left; σ = 0 ms; resembling PV inhibitory neurons), intermediate (center; σ = 10 ms; resembling non-PV inhibitory neurons), and high (right; σ = 50 ms) levels of variability (shown by variability in input position). Plot showing the depth of the inhibition strength as a function of input reliability (500 iterations; representative cases pointed by blue arrows). Highly reliable inhibitory inputs generate a sharp and strong hyperpolarization in the downstream neuron, while less reliable inhibitory inputs generate more prolonged and superficial hyperpolarization. (**B**) Same as A for 20 excitatory inputs with low (left; σ = 0 ms), intermediate (center; σ = 10 ms; resembling excitatory neurons), and high (right; σ = 50 ms) levels of variability. Plot showing the number of spikes in the downstream neuron as a function of input reliability (500 iterations; representative cases pointed by red arrows). Excitatory inputs with intermediate variability generate more spikes than those with low and high variability. (**C**) From left to right: schematic and intracellular potential evoked by the administration of 10 (left), 30 (center), and 50 (right) excitatory inputs with intermediate variability (σ = 10 ms), respectively. Plot showing the number of spikes as a function of the number of excitatory synaptic inputs for intermediate (solid black line; representative cases pointed by red arrows; 500 iterations), low, and high (dashed grey lines) levels of reliability.

To understand the network consequence of our findings, we designed a model for testing the impact of reliability on downstream neurons^27,36^, in which one Izhikevich spiking neuron^37^ receives instantaneous synaptic inputs from a population of either excitatory or inhibitory neurons, that we parametrized in terms of timing variability (Fig. 3A, B) and number of neurons (Fig. 3C). This model assumes, with respect to the previous findings, that cortical neurons are driven by common inputs^38^. Based on this assumption, a homogeneous neuronal population of highly reliable neurons will spike synchronously in response to the same input, while less reliable neurons will spike at jittered timings. This transformation from variability across trials to variability across neurons allows us to assess how spiking variability affects the downstream effects induced by multiple neurons^39^ and explores the fundamental impact of spiking variability in absence of other excitatory and/or inhibitory inputs. Although not physiological, this model provides insights about the respective contributions of excitatory and inhibitory inputs with different variability levels. Inhibitory inputs with low variability, reminiscent of the reliable spikes of PV inhibitory neurons, resulted in strong and sharp hyperpolarization with respect to highly variable inhibitory inputs (Fig. 3A), reminiscent of the more variable spikes of non-PV inhibitory neurons. Indeed, the strength of the hyperpolarization, computed as intracellular voltage reduction, progressively declined with increasing levels of variability (Fig. 3A).

Excitatory inputs with very low or high variability elicited one single spike in the downstream neuron, as opposed to the multiple spikes elicited with intermediate variability (Fig. 3B), potentially resembling the physiological properties of excitatory neurons. For this set of parameters, this is captured by an inverse u-shape relationship between excitatory input variability and number of spikes. Importantly, parameters such as time constant and strength of the synaptic inputs can critically shape this relationship, changing the relationship between variability and spike number, thus leading to a decreasing or increasing curve that reflects the properties of different cell-types^40^. Next, we varied the number of excitatory inputs (Fig. 3C). We found that excitatory inputs without any variability generated a maximum of one single spike in the downstream neuron. In contrast, for the selected parameters, temporally variable excitatory inputs elicited increasingly more spikes in the downstream neuron, proportional to the number of excitatory inputs. Importantly, the variability of the inputs determined the steepness of the relationship between number of inputs and number of spikes. Importantly, we verified that our results hold when changing the parameters of the neuronal model (Figures S3, S4, and S5), even though the effects were shifted according to the new properties of each set of parameters. This corroborates the idea that the impact of neuronal variability depends on the properties of both the presynaptic and the postsynaptic neuron. Altogether, our model suggests that low variability maximizes the impact of inhibitory inputs on downstream neurons; conversely, the impact of excitatory inputs on downstream neurons depends on the intrinsic properties of the neuron itself and of the synaptic input. Indeed, depending on these parameters, their impact can generate the maximum number of spikes in downstream neurons at high, moderate, or low synchrony levels (Figure S6).

## Discussion

Our findings indicate that spiking reliability in the timescale of few milliseconds is a cell-type specific feature related to intrinsic neuronal properties. Specifically, we found that PV neurons exhibit high reliability with respect to other cell types, particularly to excitatory neurons (Fig. 2). Furthermore, our model predicted that the low variability of inhibitory neurons allows for strong inhibition, while the higher variability of excitatory neurons allows for rate coding in downstream neurons (Fig. 3).

To our knowledge, our study is the first that examines spiking reliability of different neuronal types following direct stimulation, while previous studies examined neuronal spiking reliability following indirect stimulation, either by stimulating pre-synaptic excitatory neurons^25,40^ or by presenting sensory inputs to animals^24^. While our findings are aligned with previous studies which suggest high temporal precision in the spiking activity for inhibitory neurons, our analyses suggests that this feature of inhibitory neurons emerges also from intrinsic properties^26^, and not necessarily from synaptic mechanisms. This aligns with previous works suggesting that membrane properties, such as the density of sodium channels, enhance fast signaling, thus increasing the reliability of PV neurons^26,41^. It is noteworthy that while our study was performed in vitro, our finding that PV neurons exhibit higher reliability than other cell types aligns with previous in vivo findings^24,25^.

We have demonstrated that among inhibitory neurons, high reliability is typical of PV, but not SST (somatostatin) and VIP (vasointestinal peptide) neurons. This is in line with previous studies showing the distinct role of PV neurons in increasing reliability in responses to visual stimuli^42^ and in simulated circuits^43^. The highly synchronous spiking in PV neurons can explain their role in enhancing signal, reducing noise^23,42,44^, and functionally sculpting cortical neuronal ensembles^45^. It also highlights their role in cortical discrimination of auditory^46^, visual^42^, and somatosensory stimuli^47^. The distinct reliability properties of PV, SST, and VIP neurons suggest that they may play different functional roles. For example, among inhibitory neurons, PV neurons may gate incoming inputs by providing a timely, synchronous inhibition, while SST and VIP neurons may down-modulate cortical excitability through prolonged inhibition. This finding relates to previous works showing functional differences among inhibitory cell types, such as how PV and SST neurons exhibit different excitability levels^48^, short-term plasticity^49^, tuning properties^50^, and coordinated activity^51^.

Contrary to PV inhibitory neurons, excitatory neurons exhibit variability in their spiking activity. The reliability differences between excitatory, PV, SST, and VIP neurons suggest that different cell types may encode stimulus information through different algorithms^52,53^, with different functional significance. The spiking variability of excitatory neurons is functionally significant as it may facilitate or hinder the generation of rate codes within the cortex depending on intrinsic neuronal and synaptic properties. Our model indicates that, without this temporal variability in spiking activity, synchronous excitatory neurons would elicit a fixed firing rate in downstream neurons, regardless of the number of excitatory inputs received (see Fig. 3C)^54^. Our findings add to a large literature of models investigating the sources of variability in neuronal activity, such as stochastic neurotransmitters release^1^, resonance properties of the subthreshold membrane potential^55,56^, and refractoriness^57^.

Our analyses also indicate that human neurons exhibit lower reliability than mouse neurons (Figure S1). This finding relates to recent studies finding that mouse and human brains exhibit distinct transcriptomic^58^ and connectomic profiles^59^. The difference in spiking reliability between mice and humans may be explained, among other factors, by the larger size of human neurons^60,61^. Indeed, we found that spiking reliability covaries with the membrane time constant, which depends on the membrane surface area^62^. By affecting spiking reliability, the larger neuronal size may impact the functional properties of human neurons, potentially reducing the ability to induce sharp inhibition while promoting shallow inhibition and the integration of multiple excitatory inputs.

Our work demonstrates that spiking reliability emerges, at least in part, from intrinsic cellular properties, and that it varies among neuronal types underlying specific functions in excitatory and inhibitory neurons. Our findings provide insight into the circuit and behavioral consequences of reliability in distinct neuronal cell types. Further studies at molecular scale will reveal how reliability emerges from membrane properties and ionic channels in different neurons and neuronal compartments; in-vitro studies without synaptic blockers and in-vivo studies will illuminate the network consequences of the different reliability levels in distinct cell types.

## Materials and methods

### Metadata

Data was obtained from the Allen Cell Types datasets. Spike timings, transcriptomic cell types, and morphologic cell types were extracted from the original dataset. To classify cells in electrophysiological cell types, spike width was computed as follows. First, we averaged all the spikes evoked during the noise stimulation in one neuron. Using the so obtained “average spike”, we computed the spike width for that neuron as the duration of the spike at half of the spike amplitude. We classified units as fast and regular spiking depending on whether their spike width was below or above 400 µs, respectively^63^. The average number of repetitions was comparable across groups of neurons. Specifically, the average number of repetitions per Fs and Rs neurons was 2.35 and 2.88, respectively. The average number of repetitions per aspiny, sparsely spiny, and spiny neurons was 2.71, 2.71, and 2.94, respectively. The average number of repetitions for transcriptomic cell types is reported in Table S1. The scripts used in this paper are available at https://github.com/SimoneRusso/ReliabilityAcrossCelltypes .

### Noise stimulation protocol

In the Allen Cell Types datasets, the noise stimulation protocol was designed as follows. Two different seed values were used to generate two pink noise templates (noise template duration: 1 s; coefficient of variation: 0.2), corresponding to Noise 1 and Noise 2. To generate pink noise (i.e. noise with equal energy per octave), each noise trace was generated by summing up sine waves from 1 to 100 Hz (step 1 Hz) at random phases, each sine wave was multiplied by a coefficient proportional to the reciprocal of the square root of its frequency, hence yielding a power spectrum that falls proportionally to the reciprocal of the frequency, and the average value of the entire noise was removed from the entire noise trace^29^.Each noise template was replicated three times, concatenated, and added to a DC current of unitary amplitude to obtain a stimulus template (stimulus duration: 3 s). Each stimulus template was replicated 3 times, respectively rescaled to 75%, 100%, and 150% of the original value. These 3 traces were sorted in increasing order, separated by 5 s of recovery window, and concatenated in a single trace shown in Fig. 1A. Finally, for each neuron, this trace was rescaled according to the neuron’s rheobase value. This ensured identical noises across cells and repetitions (i.e. frozen noise) whose average value and variability were rescaled proportionally to each neuron’s rheobase. Noise autocorrelation (Figure S7) was computed on a single stimulus template of each noise using the xcorr function from Matlab 2020b (MathWorks, Natick, MA).

### Spike reliability analysis

To compute the cumulative probability density, we computed for each pair of repetitions in each neuron the latency between each spike of one repetition and the closest spike in the other repetition (with the same noise realization and intensity). These values were aggregated across sessions and neurons. Using the obtained distribution, we computed the relative cumulative density function using the ksdensity function from Matlab (function parameter: cdf).

To compute the spiking reliability (i.e. matching spikes in 1 ms window), we computed for each pair of repetitions in the same neuron the percentage of spikes occurring within 1 ms from at least one spike in each other trial. The spiking reliability of each neuron was computed as the average of the reliability values obtained from all pairs of repetitions in that neuron. This metric is resistant to low numbers of trials and is not biased by different numbers of trials, even though the estimate of the reliability may be less accurate in neurons with lower numbers of trials. To control for the firing rate, we subtracted from this value the same metric obtained from 100 shuffled data, in which the number of spikes in each session was kept constant, but their latency was randomly permuted within the stimulus time window.

We compared different cell types within mice using a Kruskal-Wallis test. Comparisons across cell types and species were carried out using a two-way ANOVA test.

### Subthreshold reliability analysis

For each neuron, we segmented the intracellular voltage recorded during the administration of 75% rheobase noise in each repetition. For each pair of repetitions in the same neuron, we computed the Spearman’s correlation between the time courses of the selected voltages. The subthreshold reliability of each neuron was then computed by averaging the Spearman’s Rho across all the pairs of repetitions within the same neuron.

### Computational model

The model was constituted by a set of synaptic inputs – parametrized in terms of number, variability, and polarity – projecting to a downstream neuron. Each synaptic input was modelled as an instantaneous current^37^ (*see Supplementary methods for model parameters and equations*), with fixed amplitude and duration across neurons. The polarity of the current was determined by the input type, being negative (hyperpolarizing) for inhibitory inputs and positive (depolarizing) for excitatory inputs. To introduce variability in the inputs’ latencies, the timing of each input was randomly sampled from a Gaussian distribution with standard deviation σ. The downstream neuron was modelled as a single-compartment Izhikevich neuron^37^ (voltage capped at 30 mV) implemented with a modified Euler scheme evaluating the v variable at twice the rate of the u variable, as in the original version of the model, which can in principle affect numerical accuracy. The Izhikevich model was used for its balance between simplicity and realistic biological properties, but analogous findings would be expected for different neuronal models. For numerical stability, we ran the model with 0.5 ms steps, according to its original implementation, and we verified that our results hold for smaller time steps.

## Electronic supplementary material

Below is the link to the electronic supplementary material.

Supplementary Material 1

Supplementary Material 2

Supplementary Material 3

## Data Availability

The Allen Cell Types dataset (http://celltypes.brain-map.org/) was published in Gouwens et al. (Nature Neuroscience, 2019).
