# Supplementary material for: Spike reliability is cell type specific and shapes excitation and inhibition in the cortex
Source: Sci Rep. 2025 Jan 2;15:350. doi: 10.1038/s41598-024-82536-y (PMC11697241; doi:10.1038/s41598-024-82536-y)
Supplement: Supplementary file 3 — Supplementary Material 3 [file 41598_2024_82536_MOESM3_ESM.docx]

**Supplementary materials for**

**“Spike Reliability is Cell Type Specific and Shapes Excitation and Inhibition in the Cortex”**

**Simone Russo^1,2^*, Garrett B. Stanley^1^, Farzaneh Najafi^3^***

^1^Wallace H Coulter Department of Biomedical Engineering, Georgia Institute of Technology and Emory University, Atlanta, GA, US

^2^Allen Institute, Brain and Consciousness Program, Seattle, WA, US

^3^School of Biological Sciences, Georgia Institute of Technology, Atlanta, GA, US

*Corresponding authors

**Figures**


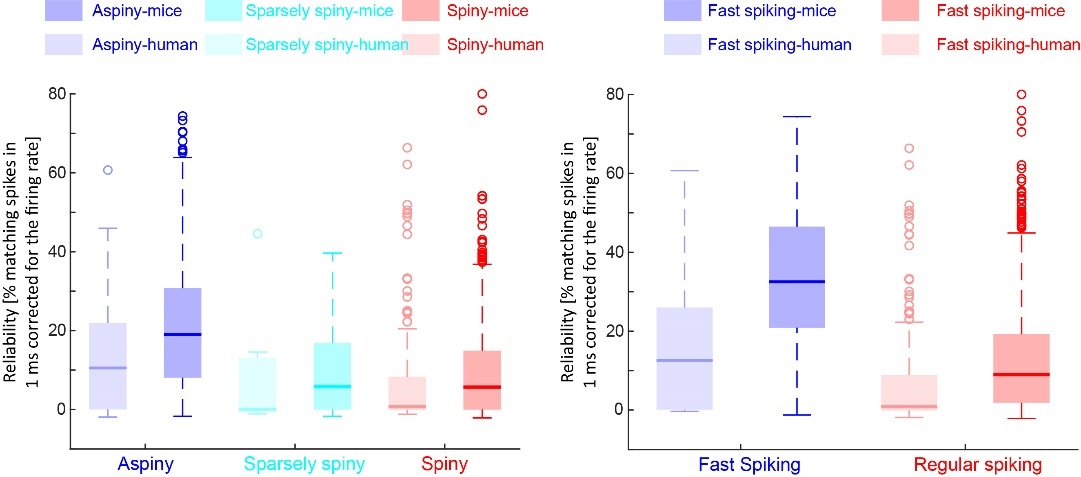


**Figure S1. Reliability across cell types and species.** Reliability (percentage of matching spikes in 1 ms window corrected for the firing rate) for morphologic (left) and electrophysiologic (right) cell types in human (shaded boxes; 319 neurons) and mice samples (solid boxes; 1532 neurons). Boxplot reports median [thick line], 25° and 75° percentile [box], maximum and minimum within 1.5 interquartile range [whiskers], and outliers [o]. Statistical details reported in Table S1.


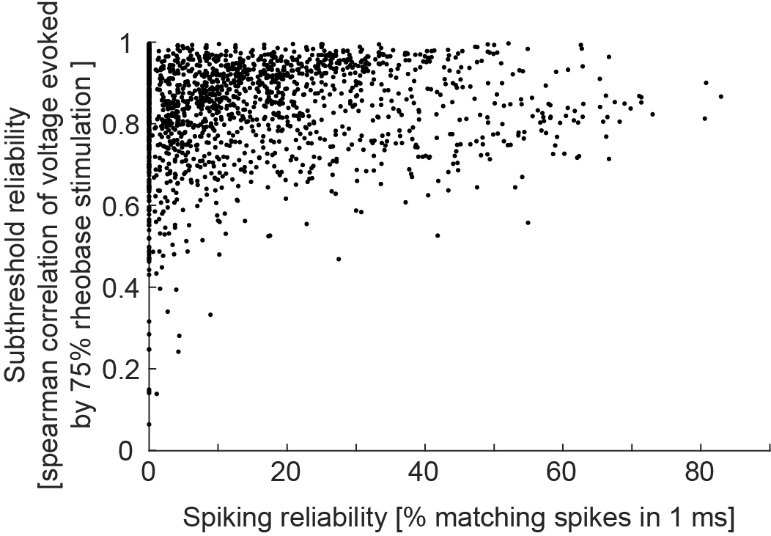


**Figure S2. Relationship between spike reliability and subthreshold reliability.** Scatter plot showing the relationship between the reliability of subthreshold fluctuations, computed as the average spearman correlation across repetitions of the noise stimulation at 75% rheobase intensity, and spiking reliability (percentage of matching spikes in 1 ms window).

**
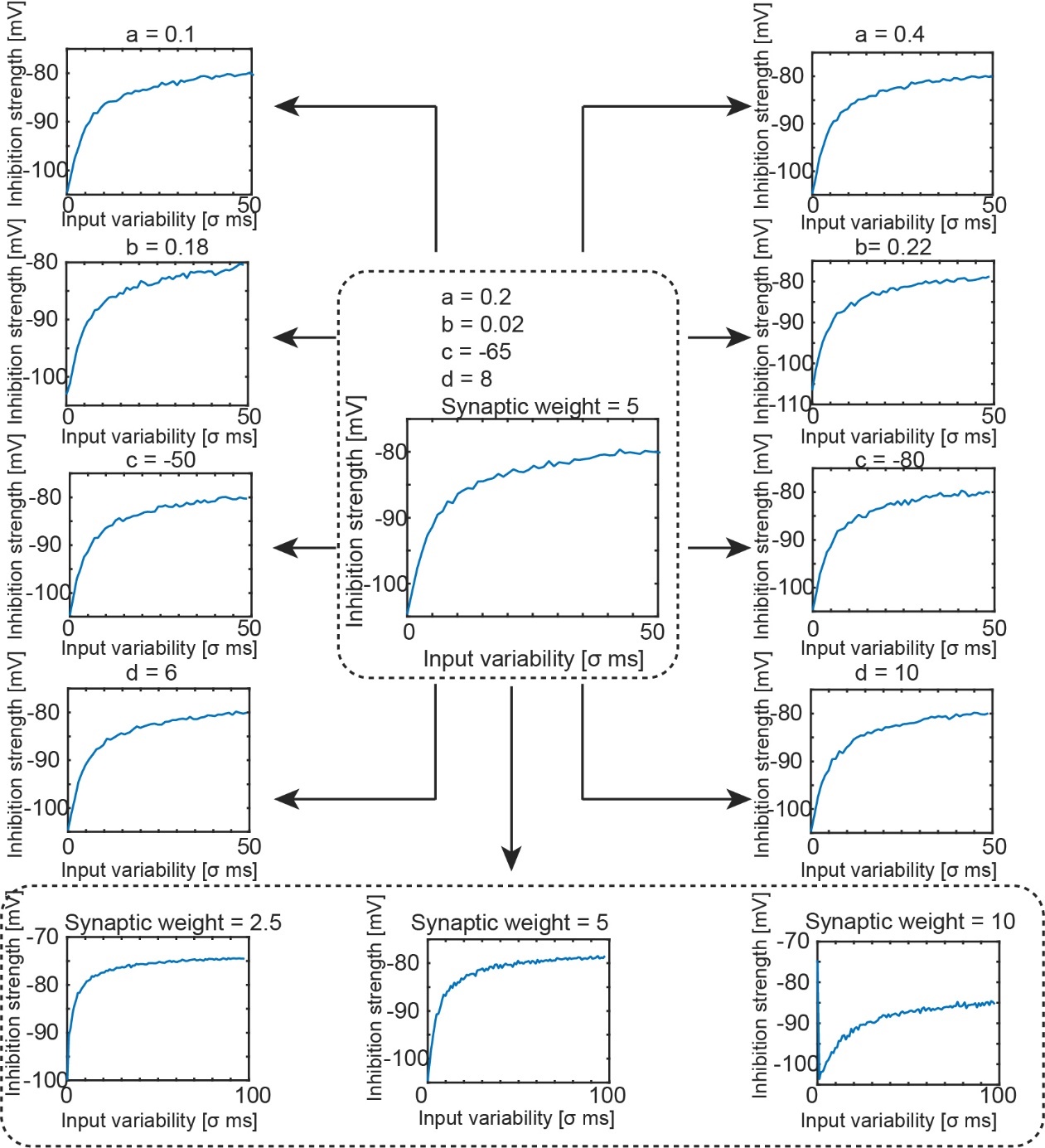
**

**Figure S3. Inhibitory inputs variability vs inhibition strength across the parameter space.** Traces showing how the relation between input variability and inhibition strength (same as Figure 3A) changes in the parameter space of the model (50 iterations per set of parameters). The central panel shows the relation between input variability and inhibition strength in the original model, while the surrounding panels show the same measure changing the model parameters. From top to bottom: parameter a, parameter b, parameter c, parameter d, synaptic weight.

**
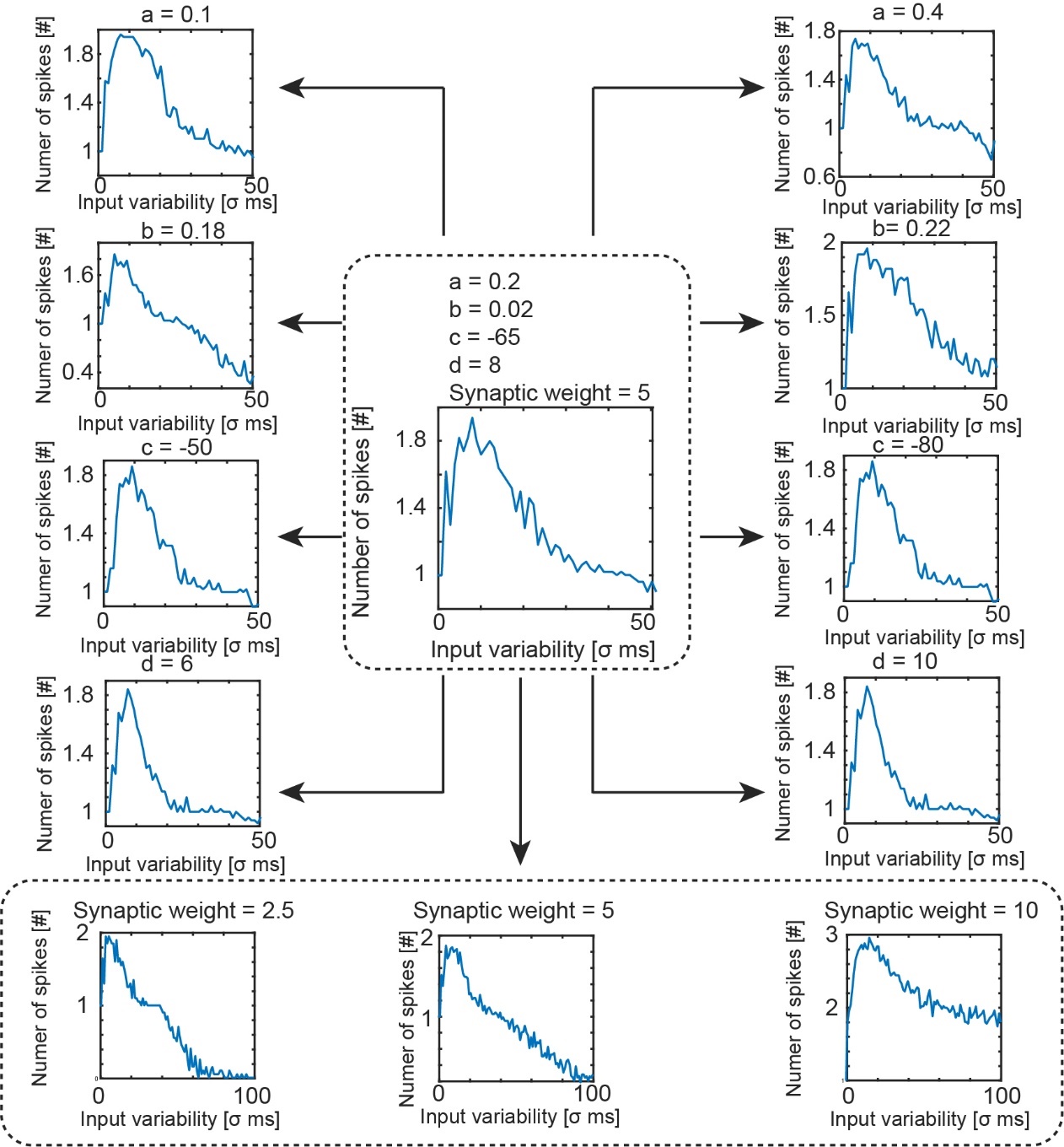
**

**Figure S4. Excitatory inputs variability vs number of spikes across the parameter space.** Traces showing how the relation between input variability and number of spikes (same as Figure 3B) in the parameter space of the model (50 iterations per set of parameters). The central panel shows the relation between input variability and number of spikes in the original model, while the surrounding panels show the same measure changing the model parameters. From top to bottom: parameter a, parameter b, parameter c, parameter d, synaptic weight.

**
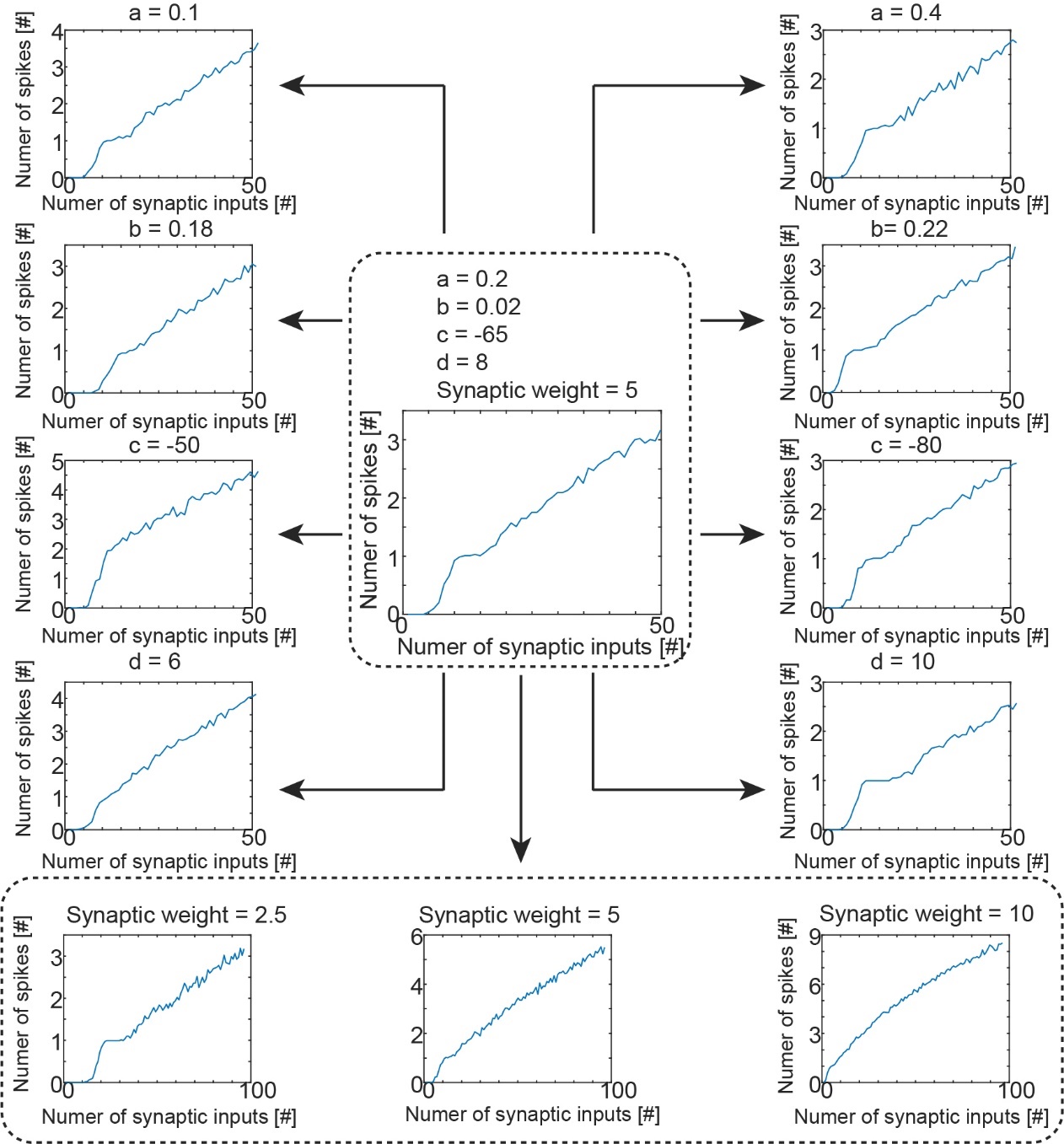
**

**Figure S5. Number of excitatory inputs vs number of spikes across the parameter space.** Traces showing how the relation between number of excitatory inputs and number of spikes (same as Figure 3C) changes in the parameter space of the model (50 iterations per set of parameters). The central panel shows the relation between input variability and number of spikes in the original model, while the surrounding panels show the same measure changing the model parameters. From top to bottom: parameter a, parameter b, parameter c, parameter d, synaptic weight.

**
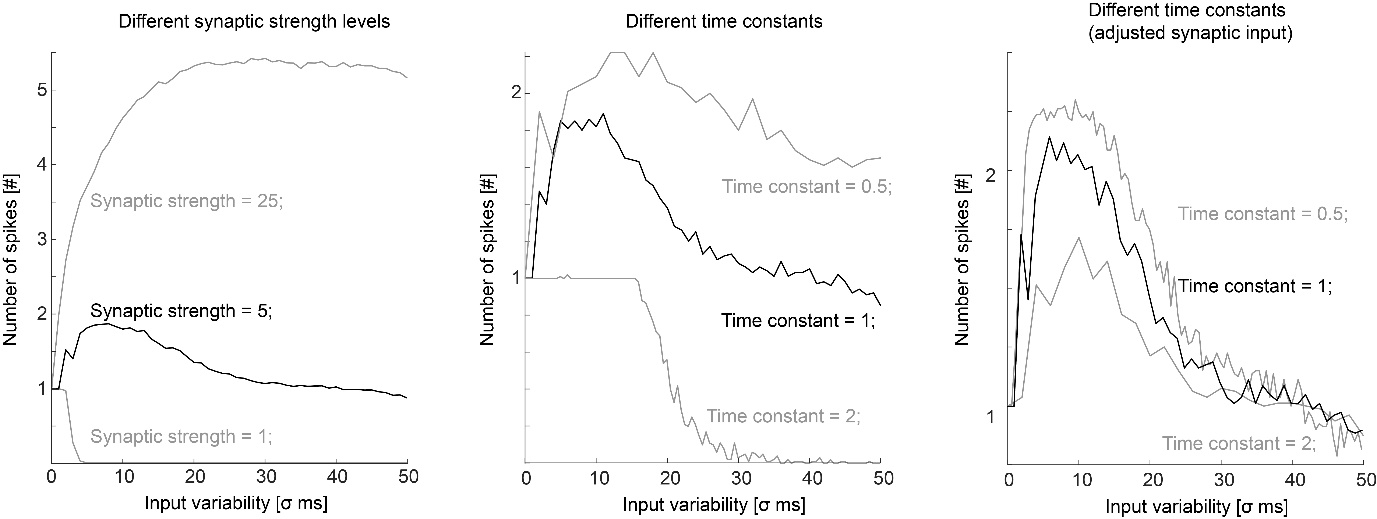
**

**Figure S6. Number of excitatory inputs vs number of spikes across synaptic strength levels and time constants.** Left: traces showing how different synaptic strength levels affect the number of spikes induced by excitatory inputs in downstream neurons, generating either increasing, decreasing or u-shaped relations. Middle: analogous traces for different time constants. Right: analogous traces for different time constants after correcting the synaptic input size for the time constant (i.e. comparable total synaptic current across time constants).


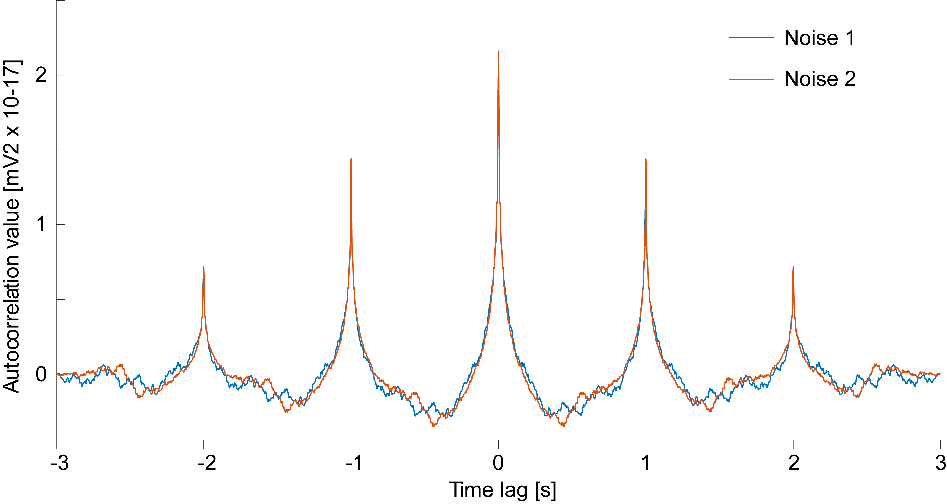


**Figure S7. Autocorrelation of frozen noise.** Traces showing the autocorrelation of the stimulus template of the two frozen noises. The five peaks reflect the three repetitions of the noise template within each stimulus template.

**Supplementary methods**

**Equations for computational modeling**

The equations used to simulate the neurons, taken from Izhikevich et al^1^, are reported below.

Differential equations:

$$v^{'}=0.04v^{2}+5v+140-u+I$$

$$u^{'}=a\left( bv-u \right)$$

After-spike resetting:

$$if v\geq30 mV, then \left\{ \begin{aligned} v\leftarrow c \\ u\leftarrow u+d \end{aligned} \right.$$

**Parameters for computational modeling**

The parameters to simulate the activity of a neuron according to the Izhikevich model taken from Izhikevich et al^1^ are reported below.

*a=[0.02];*

*b=[0.2];*

*c=[-65.^2];*

*d=[8-6];*

**Table S1**

The statistical table file (see attached file) reports the results of the Kruskal-Wallis tests and 2-way ANOVA tests (from top to bottom: Kruskal-Wallis tests for morphologic, electrophysiologic, and transcriptomic cell types in mouse neurons; 2-Way ANOVA tests for morphologic and electrophysiologic cell types). The Kruskall-Wallis and ANOVA sections of the table report, from left to right, the sum of squares, the degrees of freedom, the mean square, the chi-square, and the p value. The multiple comparisons sections of the table (i.e. multcompare), report, from left to right, the test group, the control group, the lower limit, the difference, the higher limit, and the p value. The title of the section referring to each test is highlighted in grey. For clarity, the names of the transcriptomic cell types for the relative Kruskal-Wallis test are reported in the orange column, together with their ID numbers and average number of trials per cell.

**Table S2**

The table (see attached file) reports the p-values of the Kolmogorov-Smirnov tests for the comparisons between transcriptomic cell types. The order of the cell types reported in columns and rows follows the same order specified in the orange column in Table S1.

**References**

1. Izhikevich, E. M. Simple model of spiking neurons. *IEEE Trans. Neural Netw.* **14**, 1569–1572 (2003).
